# Interactions between amphibians' symbiotic bacteria cause the production of emergent anti-fungal metabolites

**DOI:** 10.3389/fmicb.2014.00441

**Published:** 2014-08-21

**Authors:** Andrew H. Loudon, Jessica A. Holland, Thomas P. Umile, Elizabeth A. Burzynski, Kevin P. C. Minbiole, Reid N. Harris

**Affiliations:** ^1^Department of Biology, James Madison UniversityHarrisonburg, VA, USA; ^2^Department of Chemistry, Villanova UniversityVillanova, PA, USA

**Keywords:** *Batrachochytrium dendrobatidis*, emergent metabolites, amphibians, symbiotic bacteria, anti-fungal metabolites, host-associated microbial communities, interspecific competition, synergy

## Abstract

Amphibians possess beneficial skin bacteria that protect against the disease chytridiomycosis by producing secondary metabolites that inhibit the pathogen *Batrachochytrium dendrobatidis* (*Bd*). Metabolite production may be a mechanism of competition between bacterial species that results in host protection as a by-product. We expect that some co-cultures of bacterial species or strains will result in greater *Bd* inhibition than mono-cultures. To test this, we cultured four bacterial isolates (*Bacillus* sp., *Janthinobacterium* sp., *Pseudomonas* sp. and *Chitinophaga arvensicola*) from red-backed salamanders (*Plethodon cinereus*) and cultured isolates both alone and together to collect their cell-free supernatants (CFS). We challenged *Bd* with CFSs from four bacterial species in varying combinations. This resulted in three experimental treatments: (1) CFSs of single isolates; (2) combined CFSs of two isolates; and (3) CFSs from co-cultures. Pair-wise combinations of four bacterial isolates CFSs were assayed against *Bd* and revealed additive *Bd* inhibition in 42.2% of trials, synergistic inhibition in 42.2% and no effect in 16.6% of trials. When bacteria isolates were grown in co-cultures, complete *Bd* inhibition was generally observed, and synergistic inhibition occurred in four out of six trials. A metabolite profile of the most potent co-culture, *Bacillus* sp. and *Chitinophaga arvensicola*, was determined with LC-MS and compared with the profiles of each isolate in mono-culture. Emergent metabolites appearing in the co-culture were inhibitory to *Bd*, and the most potent inhibitor was identified as tryptophol. Thus mono-cultures of bacteria cultured from red-backed salamanders interacted synergistically and additively to inhibit *Bd*, and such bacteria produced emergent metabolites when cultured together, with even greater pathogen inhibition. Knowledge of how bacterial species interact to inhibit *Bd* can be used to select probiotics to provide amphibians with protection against *Bd*.

## Introduction

Amphibians are experiencing global population declines and are the most threatened vertebrate class (Stuart et al., 2004; Hoffmann et al., 2010). Indeed, 41 percent of amphibian species are threatened. Among the many factors that contribute to amphibian declines, the fungal disease chytridiomycosis, caused by the pathogen *Batrachochytrium dendrobatidis* (*Bd*), is the largest disease threat to the biodiversity of any vertebrate group (Wake and Vredenburg, 2008; Hoffmann et al., 2010). Alarmingly, disease-induced declines occur in protected, pristine environments, such as national parks, where up to 92.5 percent of critically endangered amphibian species are affected by *Bd* (Kilpatrick et al., 2010).

Amphibians have several defenses against *Bd*, including symbiotic bacteria living on their skins (Harris et al., 2006). Numerous anti-fungal bacterial species have been cultured from amphibians (Lauer et al., 2007; Woodhams et al., 2007), and their presence is associated with disease resistance (Becker and Harris, 2010). Additionally, using culture-independent techniques, diverse host-specific bacterial communities have been observed on amphibians (McKenzie et al., 2011; Kueneman et al., 2013; Loudon et al., 2013; Walke et al., 2014). One promising mitigation strategy against *Bd* is to augment the bacterial communities on amphibians' skin to increase disease resistance (Harris et al., 2009b; Bletz et al., 2013). This approach of using bacterial probiotics has been successful in laboratory experiments and one field trial (Becker et al., 2009; Vredenburg et al., 2011).

Amphibian-associated bacteria can inhibit *Bd* by producing anti-fungal metabolites (Brucker et al., 2008a,b). Several anti-*Bd* metabolites are produced from amphibian skin bacteria, such as violacein and indole-3-carboxaldehyde (I3C) from *Janthinobacterium lividum* and 2,4-diacetylphloroglucinol is produced *Lysobacter gummosus* (Brucker et al., 2008a,b). Additionally, evidence from free-living salamanders in nature suggests that the concentration of bacterially produced metabolites on their skins can be high enough to kill pathogenic fungi, such as *Bd* (Brucker et al., 2008b). Moreover, a positive correlation was found between the abundance of the amphibian symbiont *J. lividum* and violacein on red-backed salamanders; salamanders with more *J. lividum* and violacein had less morbidity due to chytridiomycosis (Becker et al., 2009).

A recent model on the assembly of beneficial microbiomes suggests that the microbiota's defensive function is a by-product of interspecific competition among microbial species (Scheuring and Yu, 2012). If microbial population densities reach the point where competition occurs and enough energy is available, defensive chemical production can be triggered that may defend the host. Without conditions of interspecific competition (pure culture), a weaker defensive function may occur. For example, bacteria cultured separately have been found to not produce anti-fungal compounds, but do produce anti-fungal compounds when cultured together (De Boer et al., 2007). In this study, we tested the hypothesis that conditions that favor interspecific competition will result in greater inhibition of *Bd* than under conditions of no interspecific competition. We hypothesized that greater inhibition of *Bd* would be caused by the production of emergent metabolites (i.e., metabolites produced in co-culture but not produced in mono-culture) that are only found with interspecific competition.

## Materials and methods

### Collection and identification of anti-Bd bacterial isolates

The bacterial isolates in this study were cultured from red-backed salamanders (*Plethodon cinereus*) that were sampled from George Washington National Forest in October, 2011. Four bacterial isolates were chosen because they inhibit *Bd* in mono-culture. The bacterial isolates were maintained in culture in 16 × 125 mm test tubes with approximately 2 mL 1% tryptone broth and incubated in a shaking incubator at 25°C. Using traditional PCR, a portion of the 16S rRNA gene was amplified using the universal 8F and 1492R primers. Reaction conditions are given in Lauer et al. (2007). The DNA was sequenced (EuroFins MWG Operon; Huntsville, Alabama), and Genious (Biomatters Limited; Auckland, New Zealand) was used to assemble the forward and reverse sequences. The GenBank database was used to identify each bacterial isolate.

### Collection of anti-Bd bacterial species' cell-free supernatants

To test bacterial isolates for *Bd* inhibition, we used a 96-well plate assay from Bell et al. (2013). Each bacterial species was re-cultured into a sterile 16 × 125 mm test tube containing 2 mL of 1% tryptone broth for 3 days and placed in an incubator at 26°C prior to the assay. Therefore, each culture had the same nutrient and environmental conditions for the same amount of time as a way to standardize growth conditions. After 3 days, cultures were noticeably turbid. To acquire cell-free supernatants (CFSs), each bacterial isolate was placed in a centrifuge tube and then homogenized with a vortexer. The bacterial isolates were then centrifuged for 5 min at 10,000 rpm. After centrifugation, the supernatants were filtered using a 0.22 μm filter (Millipore™ nitrocellulose membrane), and these solutions contained bacterial metabolites as well as other cell products. This starting solution was designated as the “high” concentration. “Medium” and “low” concentrations were 10- and 100-fold dilutions, respectively, of the “high” concentration, using 1X phosphate-buffered saline solution.

### Determination of the individual and combined effects of anti-Bd bacterial isolates on Bd inhibition

A concentration series of each isolate's CFS, alone and in combination, was used in pairwise combinations with *Bd* in a 96 well plate assay. All wells contained 50 μL of *Bd* zoospores at 2 × 10^6^ zoospores/mL in 1% tryptone broth (10^5^ zoospores/well). *Bd* zoospores were collected using the protocol of Harris et al. (2009b). To test the inhibitory effects of a single isolate, we added 25 μL CFS and 25 μL water to each well. To test the potential additive effects of two bacterial isolates grown separately, we added 25 μL of each isolate's CFS to each well. We called the latter treatment “separate then combined.” Each of these treatments was replicated five times. Positive control wells received 50 μL of sterile water without any CFSs. Negative control wells received 50 μL of heat killed *Bd* zoospores (2 × 10^6^ zoospores/mL), which were killed by placing them at 60°C for 20 min. Each of these treatments was replicated five times. Optical density (OD) readings at 490 nm were obtained at day 0 and day 5. The change in OD between days 0 and 5 indicated the amount of *Bd* growth in each well, with greater differences reflecting more *Bd* growth.

### Determination of the effects of interspecific competition on Bd inhibition

To assess the effects of interspecific bacterial competition on *Bd* inhibition, bacterial species were cultured in paired combinations for 3 days prior to assays with *Bd*. For these co-culture treatments, 5 μL of the original culture of each bacterial species was placed into a test tube containing 2 mL of 1% tryptone broth. Mono-cultures and additional “separate then combined” assays were done using the procedures stated above as comparisons. Bacterial culturing conditions were the same as the previous assay. The co-culture wells contained 50 μL of the CFS from paired combinations of bacterial species, and each treatment was replicated five times.

### Detection and isolation of emergent metabolites

CFC's of monocultures and co-cultures of all isolates were extracted with ethyl acetate according to the method of Brucker et al. (2008a). The *Bacillus* sp. and *Chitinophaga arvensicola* combination was focused on since they resulted in the greatest *Bd* inhibition in co-culture. The resulting crude extracts were evaporated to dryness *in vacuo*, reconstituted in 0.75 mL methanol, and analyzed by reversed-phase, high performance liquid chromatography (HPLC, 25 μL injection) using a Shimadzu LC-20 liquid chromatograph equipped with an ACE C18 column (3 μm, 150 × 4.6 mm) according to the method of Umile et al. (2014). Comparison of the chromatograms showed the appearance of at least six metabolites in the co-culture that were not present in either monoculture (**Figure 3**). Metabolite peaks that were observed in co-cultures, but not present in either of the two individual cultures, were identified as emergent metabolites. These metabolites were isolated by semi-preparative HPLC [ACE C18 column (5 μm, 250 × 10 mm)] and assayed for bioactivity toward *Bd* (see below). To test this, each fraction was suspended in 5% DMSO solution, to facilitate the dissolution of metabolites. 10 μL of each metabolite solution was added to 40 μL of a *Bd* zoospore solution resulting in a final concentration of 10^6^ zoospores/mL. The final concentration of each metabolite was ≈2 μg/mL. In addition, tests were performed without the use of DMSO as a co-solvent with low concentrations of each metabolite that were not quantified. These tests suggested that the compound eluting at 13.48 min (“Compound 3”) was the most inhibitory (**Figure 4**).

### Characterization and identification of “compound 3”

An inhibitory compound eluting at 13.48 min (“Compound 3”) was isolated using the semi-preparative HPLC described above and analyzed by ^1^H NMR and liquid chromatography-mass spectrometry (LC-MS). ^1^H NMR spectra were measured with a 300 MHz Varian spectrophotometer. UV-Vis measurements utilized a Shimadzu SPD-M20A diode array detector, and mass spectrometry used an Applied Biosystems SCIEX API 2000 triple quadrupole mass spectrometer operating in positive electrospray ionization mode.

### Bioassay with metabolite and Bd

The IC_50_ for the emergent metabolite, tryptophol, against *Bd* strain JEL310 was determined using a 96 well plate method assay previously developed by Brucker et al. (2008a).

### Statistical analyses

Analyses of variance (ANOVA) were used to test for the main effects of species' CFS on *Bd* inhibition. A significant interaction effect indicated that either synergy or antagonism between species' CFS was present. Dunnett's test was used for a posteriori tests to compare treatments to controls, and Tukey's test was used to compare each treatment to each other to identify an interaction effect (synergy or antagonism) when one was present.

## Results

### Bacterial identification

Isolates were determined to be a *Bacillus* sp., *Janithobacterium* sp., *Pseudomonas* sp., and *Chitinophaga arvensicola* (Table 1; sequences Genbank accession #'s KJ923801, KJ923802, KJ923803 and KJ923804 respectively). The genera of all four isolates were represented within the Illumina dataset presented in Loudon et al. (2013). In addition, OTUs within the family Pseudomonadaceae were within the core bacterial community and *Janthinobacterium lividum* was highly prevalent on red-backed salamanders.

**Table 1:** **Identities of anti-*Bd* bacterial isolates used in inhibition assays**.

**Identity**	**% match**	***E*-value**	**Accession number**
*Bacillus* sp.^*^	100	0	KJ923801
*Pseudomonas* sp.^*^	100	0	KJ923802
*Janthinobacterium* sp.^*^	99	0	KJ923803
*Chitinophaga arvensicola*	99	0	KJ923804

* *Isolates were only identified to the Genus level*.

### “Separate then combined” assays

To determine the effect of combined bacterial isolates on *Bd* inhibition we used a “separate then combined” assay where isolates were first grown in mono-culture, and then combined together to challenge *Bd*. The six combinations of the four bacterial species' CFSs demonstrated several effects on the growth of *Bd* in comparison to the positive *Bd* control (Supplementary Table 1). The “separate then combined” results for *Pseudomonas* sp. and *Chitinophaga arvensicola* were not included due to failure of the positive controls; this resulted in 45 “separate then combined” combinations. Of the 45 combinations assayed, some produced an additive effect when assayed with *Bd*, some synergistically inhibited the growth of *Bd* in comparison to the positive control, and others had no effect on *Bd* growth.

**Figure 1:**
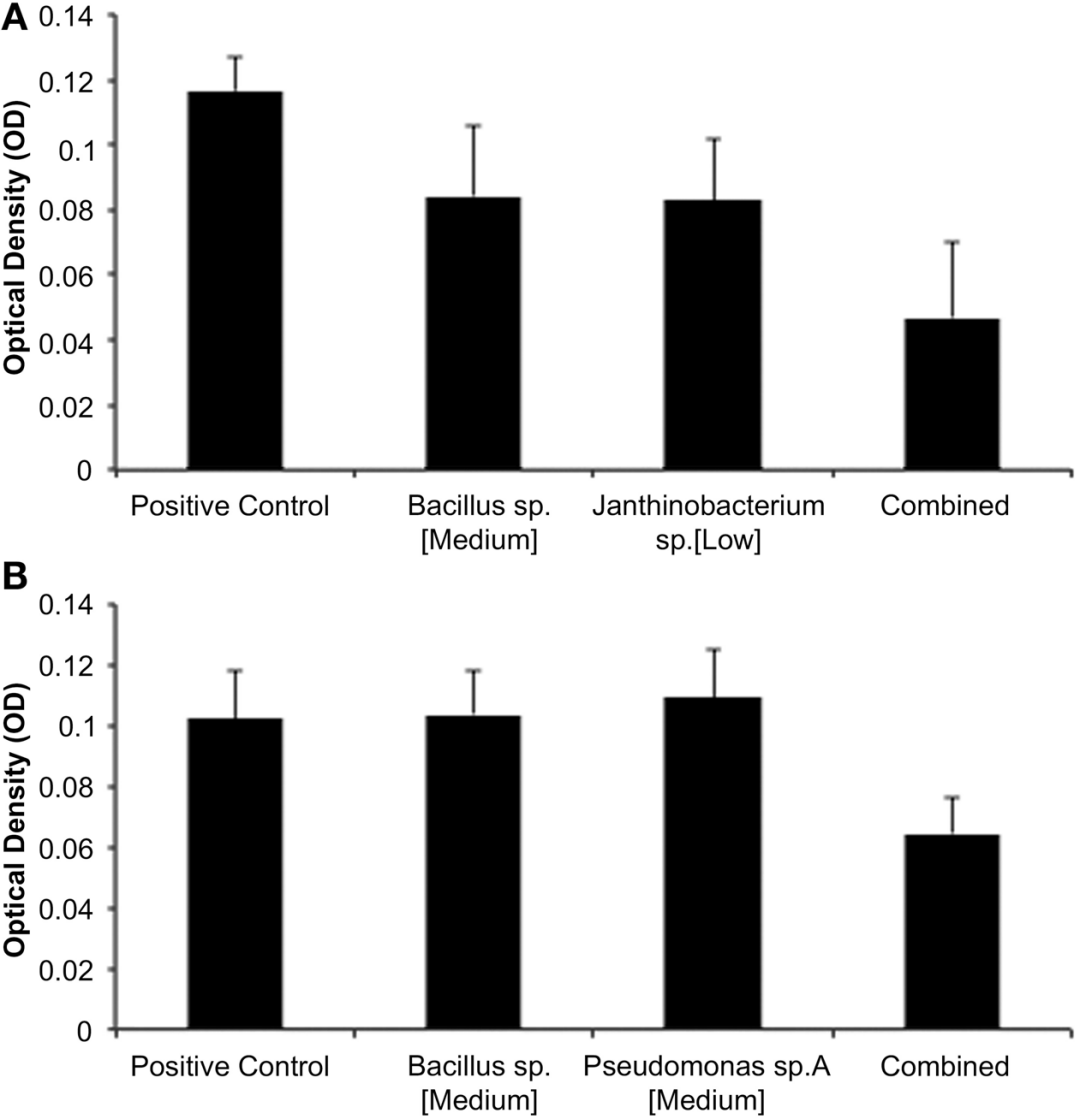
**(A,B)** Representative results from “separate then combined” assays. **(A)** Additive *Bd* inhibition with a medium concentration of *Bacillus* and *Janthinobacterium* (ANOVA- *Bacillus p* = 0.0011; *Janthinobacterium p* = 0.0008; Interaction *p* = 0.8277). **(B)** Synergisytic inhibition of *Bd* with a medium concentration of *Bacillus* and *Pseudomonas* sp. *A* (ANOVA- *Bacillus p* = 0.0512; *Pseudo A p* = 0.0239; Interaction *p* = 0.0025).

Of the total of 45 isolate-CFS concentration combinations, 19 of these exhibited an additive relationship when assayed against *Bd*. These pairs showed an effect on *Bd* growth equal to the sum of the effects of the CFSs of each species. One example of an additive relationship was between the medium concentration of *Bacillus* sp. CFS and the low concentration of *Janthinobacterium* sp. CFS (Figure 1A). There was a tendency of high concentration combination of both isolates to have additive effects as well.

There were 19 isolate-CFS concentration combinations that inhibited *Bd* synergistically. These combinations inhibited *Bd* with a greater total effect than the additive relationship. Concentration combinations were identified as synergistic if their statistical interactions were significant. For example, the combinations of the medium concentration of *Bacillus* sp. CFS with the medium concentration of *Pseudomonas* sp. resulted in synergy (Figure 1B).

### Co-culture assays

**Figure 2:**
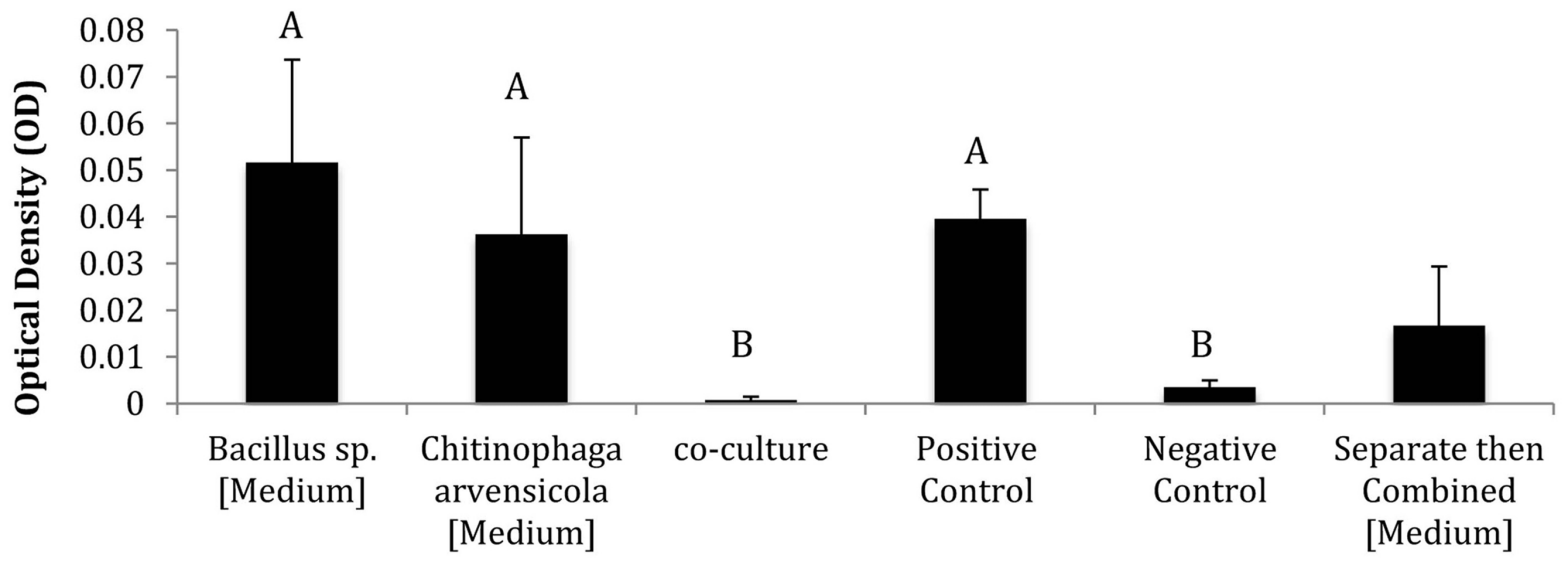
**Representative results of co-culture vs. mono-culture assay using *Bacillus* sp. and *Chitinophaga arvensicola* (Different letters indicate that treatments are significantly different at *p* < 0.05 level using Tukey's test)**. The “separate then combined” results for this isolate combination are also included as a comparison.

The CFSs from co-cultured bacterial isolates showed greater inhibition of *Bd* than the “separate then combined” CFSs in some cases. Out of the six co-culture combinations, four resulted in synergistic inhibition of *Bd*, one resulted in additive inhibition of *Bd*, and one resulted in synergistic facilitation of *Bd* (Supplementary Table 2). The co-cultured combinations of *Bacillus* sp./*Janthinobacterium* sp., *Bacillus* sp./*Pseudomonas* sp., *Bacillus* sp./*C. arvensicola* (Figure 2) and *Janthinobacterium* sp./*C*. synergistically inhibited *Bd*. The co-culture combination of *Pseudomonas* sp./*C. arvensicola* additively inhibited *Bd*. Lastly, the combination *Pseudomonas* sp./*Janthinobacterium* sp. synergistically facilitated *Bd*.

The “separate then combined” assays that were repeated for a comparison with the co-culture assays sometimes differed from initial “separate then combined” trials. For the *Bacillus* sp./ *Pseudomonas* sp. combination, the “separate then combined” treatments from the two different trials were significantly different between trials. These comparisons were performed after standardizing the OD values by the positive controls from the two different assays.

### Emergent metabolites

**Figure 3:**
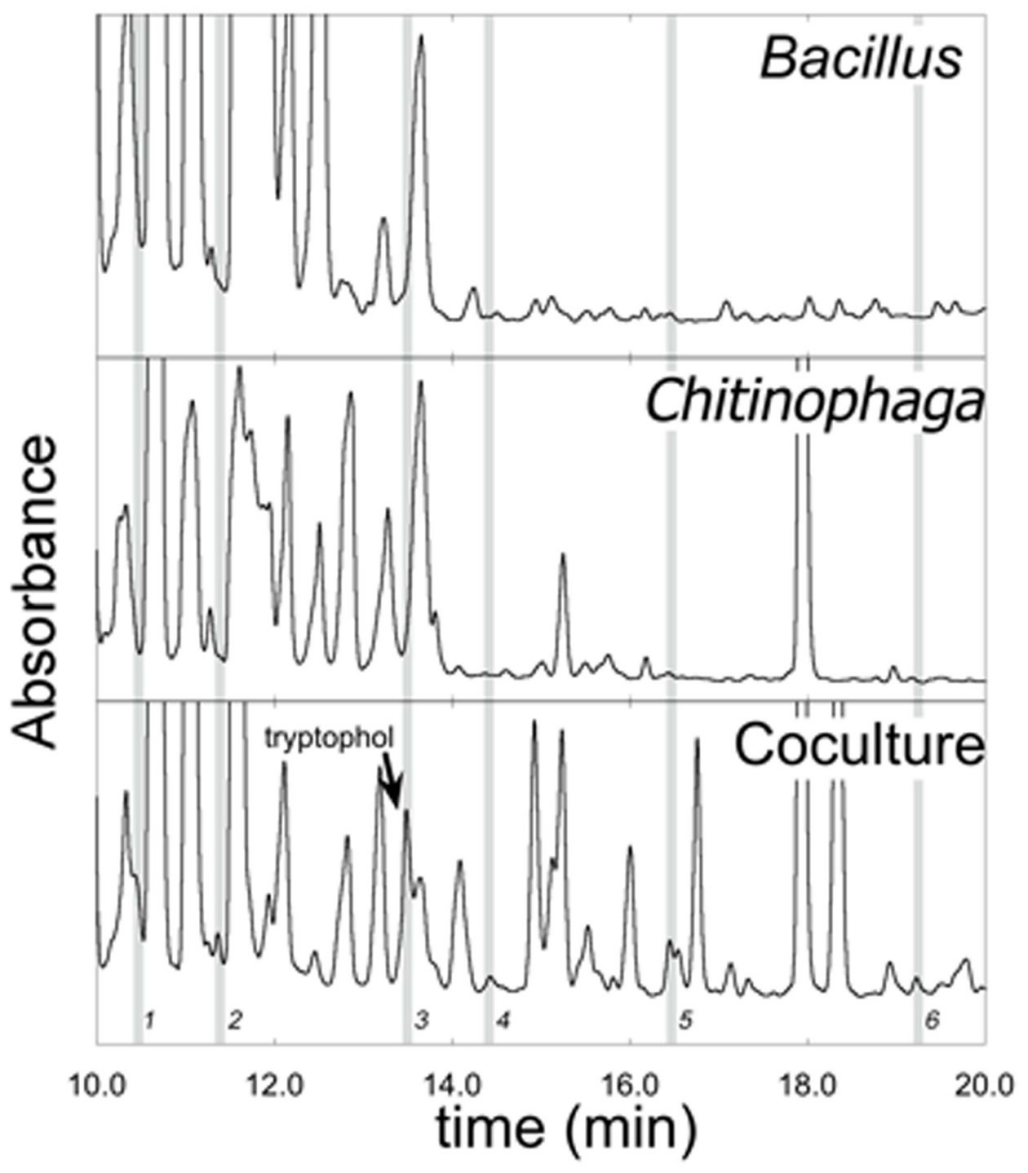
**LC-MS chromatograms of ethyl acetate extracts from monocultures of *Bacillus* and *Chitinophaga arvensicola* and a co-culture of the two**. Gray bars indicate emergent metabolites found in the co-culture that were not present in either monoculture. (Absorbance axes not to common scale.) Metabolites are labeled 1–6; metabolite 7 has a retention time greater than what is shown in this figure and is therefore not present.

**Figure 4:**
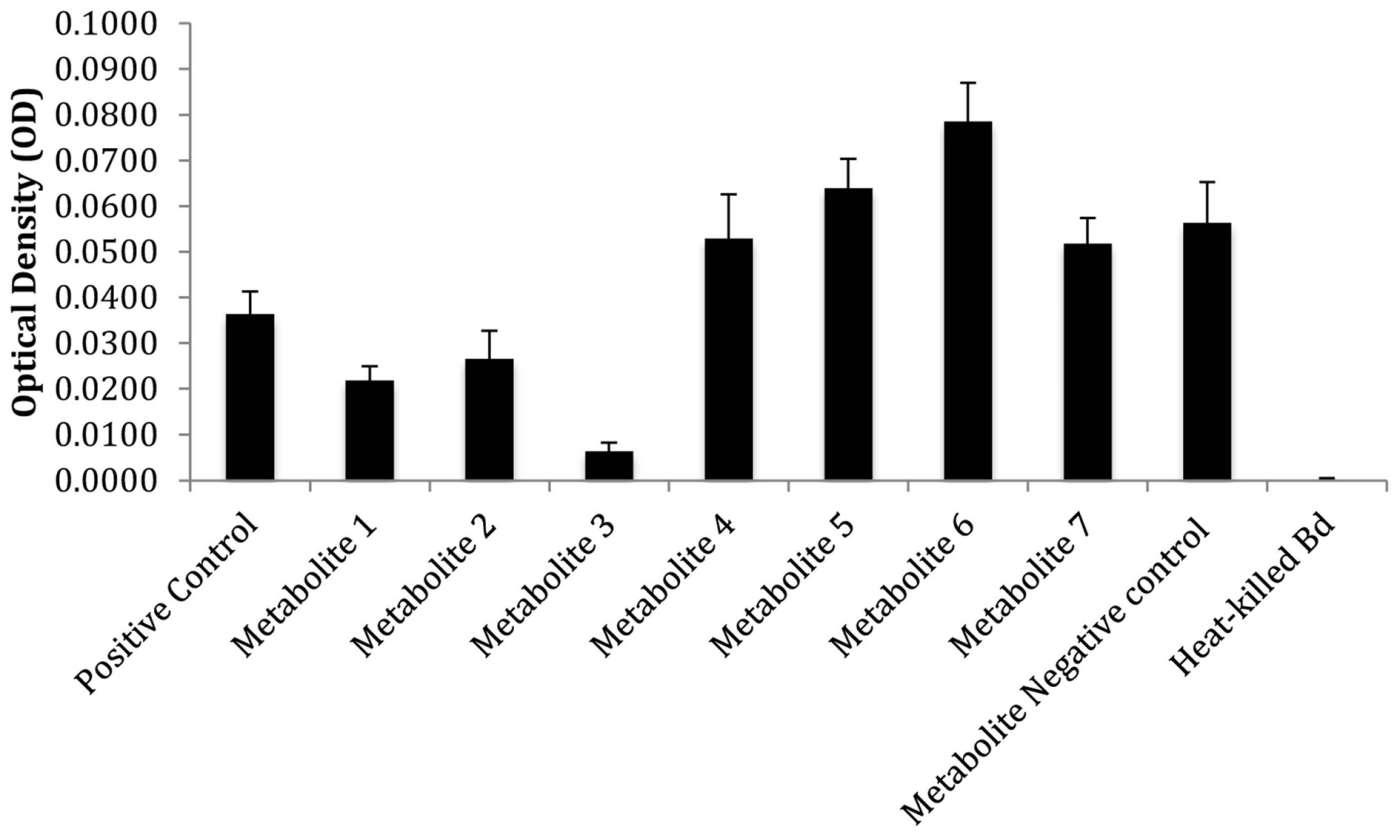
**Inhibitory properties of emergent metabolite fractions 1–7 at low unquantifiable concentrations**. An additional control, “metabolite negative control,” was also included, which was a fraction that contained no metabolites. The bars reflect standard deviation.

The co-culture between *Bacillus* sp. and *Chitinophaga arvensicola* produced seven isolated emergent metabolites (Figure 3) that were all inhibitory to *Bd* growth at an approximate concentration of 2 μg/mL. An additional test that did not use DMSO as a co-solvent and using low unquantified metabolite concentrations revealed that three compounds were inhibitory, with Compound 3 being the most inhibitory (Figure 4).

**Figure 5:**
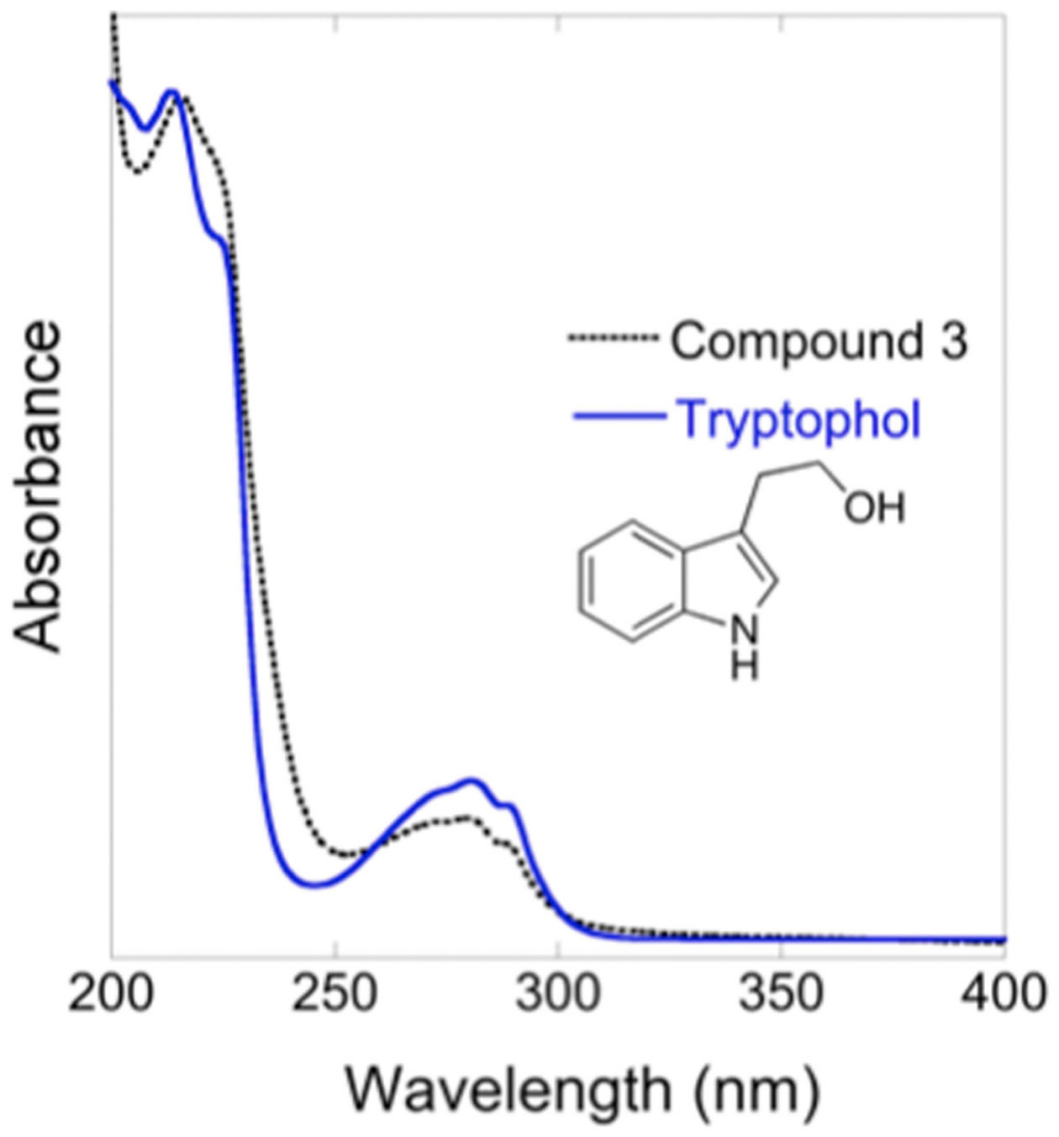
**UV-Vis spectra of isolated Compound 3 and an authentic tryptophol standard**.

**Figure 6:**
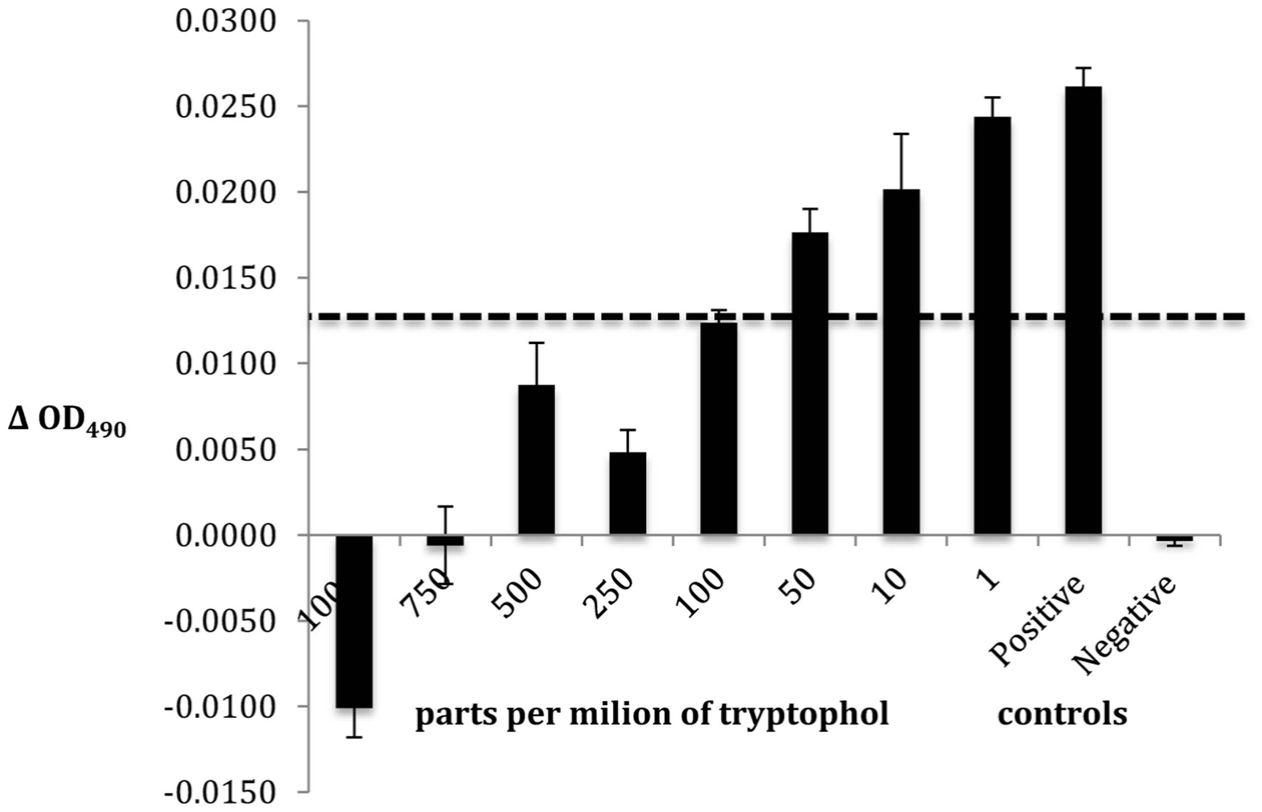
***Bd* challenge assay to determine the IC_50_ of the emergent metabolite tryptophol**. The bars reflect standard deviation.

Compound 3 was identified as tryptophol by multiple chemical methods (UV-Vis spectroscopy, mass spectrometry, and ^1^H NMR spectroscopy). Accordingly, isolated Compound 3 and an authentic tryptophol standard co-elute (13.48 min) and share a characteristic indole chromophore with peaks at 280 and 289 nm (Figure 5). Additionally, ESI-MS of a tryptophol standard and Compound 3 both show a characteristic 144 m/z peak corresponding to an [M—H_2_O]^+^ ion; finally, the ^1^H NMR spectrum of isolated Compound 3 matches that of authentic tryptophol. Authentic tryptophol inhibited *Bd* (Figure 6) and displayed an IC_50_ of ~100 ppm (~600 μM).

The metabolite profiles for *Pseudomonas* sp. and *Janthinobacterium* sp. were also examined since this combination resulted in *Bd* facilitation. *Janthinobacterium* sp. had three metabolites that were present in the mono-culture that were not present in the co-culture; therefore, *Bd* was challenged with a different suite of metabolites than when challenged with the mono-cultures.

## Discussion

Combinations of bacterial isolate's CFSs exhibited several different relationships in the inhibition of *Bd*, demonstrating that the isolates, and the concentrations of an isolate's CFSs, are important factors in *Bd* inhibition. In some cases, the inhibition caused by two isolates CFSs had an additive effect on *Bd* growth inhibition. Other combinations inhibited *Bd* growth synergistically. Some combinations did not demonstrate any effect on *Bd* growth.

The *in vitro* model of combining CFSs can represent spatially separated isolates on amphibian skin whose secreted metabolites combine in the interstices between them. The different relationships among the pairwise combinations between the four isolates in high, medium and low concentrations demonstrate the variability of bacterial interactions that are likely found in nature. It is unknown how isolates are distributed on amphibian skins, much less their relative population densities, but it is likely to be variable even on a single host individual. In an additive relationship, the metabolites of both bacterial isolates are contributing equally to the inhibition of *Bd* growth. Synergistic relationships may be the result of anti-fungals with different modes of action (Ghannoum and Rice, 1999). As a hypothetical example, in our system a metabolite from the first bacterial isolate may puncture the cell wall of *Bd*, whereas the metabolites from the second bacterial isolate may inhibit translation.

Emergent metabolites were identified that were present in bacterial co-cultures, but not present in bacterial mono-cultures. CFSs from co-cultures were often more inhibitory of *Bd* than combining CFSs from mono-cultures. Co-culturing is a model of isolates on the skins that are in the same or close spatial location and are competing with each other by starting to produce additional inhibitory metabolites, and these new metabolites are one reason for greater inhibition of *Bd*. Furthermore, emergent metabolites may be synergistically interacting with the metabolites produced in mono-culture and other emergent metabolites to enhance their collective killing power. We determined that these emergent metabolites are anti-*Bd* and determined the identity and IC_50_ of the most inhibitory metabolite, tryptophol. Although it is inhibitory, it is much less potent than previously identified anti-*Bd* metabolites such as violacein (Brucker et al., 2008b). Tryptophol has been found to be produced by fungi such as *Ceratocystis adipose* (Guzmán-López et al., 2007), *Candida albicans* (Lingappa et al., 1969), *Pythium ultimum* (Rey et al., 2001), *Drechslera nodulosum* (Sugawara and Strobel, 1987), and *Zygosaccharomyces priorianus* (Rosazza et al., 1973). The mechanism that tryptophol uses to inhibit *Bd* is unknown. We hypothesize that these emergent metabolites are interacting to cause the greater *Bd* inhibition found in the co-culture assays. The strong inhibition of *Bd* caused by the co-culture CFSs supports the hypothesis that interspecific competition led to greater *Bd* inhibition than intraspecific competition alone (De Boer et al., 2007; Scheuring and Yu, 2012). Furthermore, it suggests that competing microbial species in amphibian skins would enhance disease protection.

One case of facilitation occurred in co-culture between *Pseudomonas* sp. and *Janthinobacterium* sp. This result is intriguing since when the bacteria were grown separately and then combined, they did not result in facilitation. Previous work using a similar protocol to determine bacteria's effect on *Bd* found facilitation of *Bd* growth by some isolate's CFSs (Bell et al., 2013). *Pseudomonas* can produce a number of antifungal metabolites and *Janthinobacterium* has been widely studied as an anti-*Bd* bacterium, so facilitation of *Bd* was surprising. In our study, the co-culture lacked metabolites that were found in the *Janthinobacterium* mono-culture. This shift in metabolites may have led to the facilitation of *Bd*.

When the “separate then combined” assays were repeated, the results differed to some extent between trials. In particular, synergy between certain pairs of isolates in the first experiment was not always found in the second experiment. A possible reason for this is that isolates were under somewhat different densities in culture due to stochastic variation. As a result, isolates produced different concentrations of metabolites that then interacted differently when combined. An additional reason may be the age of the media used or the use of different batches of media that had slight chemical differences. Also, it is possible that mutation in laboratory stocks led to different outcomes in the assays. However, all of the results demonstrate that additive and synergistic interactions occur among amphibians' skin bacteria, which depend on a variety of factors, including bacterial species identify and chemical concentration.

The inhibitory interactions between different bacterial species are not the only chemical interactions that inhibit *Bd* within the amphibian skin system. Myers et al. (2012) found synergistic interactions between the antimicrobial peptides (AMPs) from the skin of the frog, *Rana muscosa*, and the known anti-*Bd* metabolite 2,4-diacetylphloroglucinol (2,4-DAPG), which is produced by the bacterium *Pseudomonas fluorescens*. It is likely that AMPs interact with other bacterially-produced metabolites to inhibit *Bd*, especially since we found that bacteria produce a high richness of metabolites. Bacteria also produce AMPs that may inhibit *Bd*, although we focused on smaller metabolites that are produced by bacteria.

Our results support the hypothesis that competition between different bacterial species causes greater *Bd* inhibition. Bacterial species compete through the production of defensive compounds, which leads to their competitive dominance. Therefore, the host obtains a defensive benefit as a by-product of microbial competition. Results from theory suggest that beneficial, antibiotic-producing bacteria are favored over bacteria that do not produce such compounds as long as the host supplies adequate resources for antibiotic production (Scheuring and Yu, 2012). Although we expect that competition has driven the production of emergent metabolites and an overall greater inhibitory function, competition was not measured, and it is possible that other interactions, such as cooperation, led to our results.

This work reveals the complexity of microbial interactions and emphasizes the importance of considering such interactions when developing a bacterial probiotic as a conservation strategy. Furthermore, our results suggest that a bacterial probiotic with multiple bacterial species is more promising than a bacteria probiotic with a single bacterial species. Although a single trial to test bacterial probiotics with multiple bacterial species was unsuccessful on amphibians (Bletz et al., 2013), more work is needed to fully assess the potential of such therapy. Probiotic trials with a single isolate have been successful in some cases (Harris et al., 2009a,b), but not in others (Becker et al., 2011; Bletz et al., 2013). This variation could be explained by the probiotic interacting synergistically with the resident microbiota to inhibit *Bd* in the successful cases, but not synergistically inhibiting *Bd* or even facilitating *Bd* the unsuccessful cases. Bacterial probiotics with multiple bacterial species have been successful in mouse intestines (Lawley et al., 2012). In this study, the successful combination of bacteria used in the probiotic was selected by trial and error. This strategy was successful, however not efficient. It is likely important to choose bacteria that will compete interspecifically and produce more inhibitory metabolites. We propose choosing bacteria that display additive or synergistic relationships when in combination with other isolates over a range of concentrations and be highly inhibitory when co-cultured. If a bacterial isolate does not have an effect on *Bd* growth and does not produce any effect when combined with a second bacterial isolate, then it should be excluded from further consideration as part of a probiotic solution. To test whether the addition of multiple probiotics on amphibians worked *in vivo*, the defensive function of the mucosome could be measured, which would determine if the chemicals on an amphibian are anti-fungal prior to infection by a pathogen (Woodhams et al., 2014). Our results provide support for the use of a co-culture method in probiotic development that may lead to a more effective probiotic solution.

In conclusion, we found that metabolites produced by amphibians' skin bacteria in monoculture can be combined, and they can interact synergistically with each other to inhibit *Bd*. In addition, we found that when these bacteria were co-cultured they were often even more inhibitory to *Bd*, and they produced emergent metabolites that were not made by the bacteria when in monoculture. Furthermore, we identified the emergent metabolite tryptophol, which was the most active emergent metabolite against *Bd*. These results will aid in developing combination of probiotics to mitigate the amphibian disease chytridiomycosis, in addition to contributing to our knowledge of bacterial and chemical interactions.

### Conflict of interest statement

The authors declare that the research was conducted in the absence of any commercial or financial relationships that could be construed as a potential conflict of interest.
