# Supplementary material for: Interactions between amphibians' symbiotic bacteria cause the production of emergent anti-fungal metabolites
Source: Front Microbiol. 2014 Aug 21;5:441. doi: 10.3389/fmicb.2014.00441 (PMC4139739; doi:10.3389/fmicb.2014.00441)
Supplement: Supplementary file 1 [file DataSheet1.DOCX]

Supplementary Table 1. Comparison of means of “separate then combined”. An ANOVA was used to determine if isolate combinations resulted in additive inhibition of *Bd*, synergistically inhibition of *Bd* or no effect. Low, med, and high refer to concentrations as described in the text. Percent of *Bd* growth when grown each separate isolate alone and combined with standard deviation are included.

| Combination | |  | P value | | |  | % Bd Growth | | |
| --- | --- | --- | --- | --- | --- | --- | --- | --- | --- |
| Treatment 1 | Treatment 2 | N | T1 main effect | T2 main effect | Interaction | Biological effect | T 1 | T2 | Separate then combined |
| Bacillus sp. [low] | Pseudomonas sp. [low] | 5 | 0.0063 | 0.4941 | 0.0185 | Synergy | 97±13 | 120±7 | 85±16 |
| Bacillus sp. [low] | Pseudomonas sp. [med] | 5 | 0.4692 | 0.0661 | 0.2759 | No effect | 97±13 | 107±16 | 122±26 |
| Bacillus sp. [low] | Pseudomonas sp. [high] | 5 | 0.9685 | 0.4598 | 0.7283 | No effect | 97±13 | 89±7 | 93±39 |
| Bacillus sp. [med] | Pseudomonas sp. [low] | 5 | 0.0019 | 0.8751 | 0.0011 | Synergy | 101±14 | 120±7 | 80±9 |
| Bacillus sp. [med] | Pseudomonas sp. [med] | 5 | 0.0041 | 0.0239 | 0.0025 | Synergy | 101±14 | 107±16 | 63±11 |
| Bacillus sp. [med] | Pseudomonas sp. [high] | 5 | 0.0512 | 0.0003 | 0.0286 | Synergy | 101±14 | 89±7 | 66±5 |
| Bacillus sp. [high] | Pseudomonas sp. [low] | 5 | <.0001 | 0.092 | 0.0302 | Synergy | 67±4 | 120±7 | 63±13 |
| Bacillus sp. [high] | Pseudomonas sp. [med] | 5 | <.0001 | 0.2054 | 0.0468 | Synergy | 67±4 | 107±16 | 37±29 |
| Bacillus sp. [high] | Pseudomonas sp. [high] | 5 | <.0001 | 0.1244 | 0.6811 | Additive | 67±4 | 89±7 | 59±17 |
| Bacillus sp. [low] | Janthinobacterium sp. [low] | 5 | 0.0924 | 0.0013 | 0.6475 | Additive | 85±22 | 71±16 | 62±7 |
| Bacillus sp. [low] | Janthinobacterium sp. [med] | 5 | 0.0021 | 0.0002 | 0.1558 | Additive | 85±22 | 78±7 | 42±18 |
| Bacillus sp. [low] | Janthinobacterium sp. [high] | 5 | 0.0613 | <.0001 | 0.0003 | Synergy | 85±22 | 16±6 | 53±7 |
| Bacillus sp. [med] | Janthinobacterium sp. [low] | 5 | 0.0011 | 0.0008 | 0.8277 | Additive | 72±18 | 71±16 | 40±19 |
| Bacillus sp. [med] | Janthinobacterium sp. [med] | 5 | <.0001 | 0.0003 | 0.7458 | Additive | 72±18 | 78±7 | 47±7 |
| Bacillus sp. [med] | Janthinobacterium sp. [high] | 5 | 0.0074 | <.0001 | 0.0317 | Synergy | 72±18 | 16±6 | 0±8 |
| Bacillus sp. [high] | Janthinobacterium sp. [low] | 5 | 0.0156 | 0.0347 | 0.2382 | Additive | 68±22 | 71±16 | 59±23 |
| Bacillus sp. [high] | Janthinobacterium sp. [med] | 5 | <.0001 | <.0001 | 0.0334 | Synergy | 68±22 | 78±7 | 19±6 |
| Bacillus sp. [high] | Janthinobacterium sp. [high] | 5 | 0.0006 | <.0001 | 0.1342 | Additive | 68±22 | 16±6 | 1±2 |
| Bacillus sp. [low] | Chitinophaga arvensicola [low] | 4 | 0.1478 | 0.2046 | 0.3032 | No effect | 105±13 | 76±34 | 102±10 |
| Bacillus sp. [low] | Chitinophaga arvensicola [med] | 4 | <.0001 | <.0001 | <.0001 | Synergy | 105±13 | 103±6 | 23±7 |
| Bacillus sp. [low] | Chitinophaga arvensicola [high] | 4 | 0.2103 | <.0001 | 0.5599 | Additive | 105±13 | 51±4 | 64±18 |
| Bacillus sp. [med] | Chitinophaga arvensicola [low] | 4 | 0.2079 | 0.1106 | 0.7925 | No effect | 82±51 | 76±34 | 49±27 |
| Bacillus sp. [med] | Chitinophaga arvensicola [med] | 4 | 0.0133 | 0.2118 | 0.1341 | Additive | 82±51 | 103±6 | 42±16 |
| Bacillus sp. [med] | Chitinophaga arvensicola [high] | 4 | 0.4543 | 0.0176 | 0.7216 | Additive | 82±51 | 51±4 | 49±34 |
| Bacillus sp. [high] | Chitinophaga arvensicola [low] | 4 | 0.1127 | 0.1208 | 0.9019 | No effect | 75±18 | 76±34 | 54±36 |
| Bacillus sp. [high] | Chitinophaga arvensicola [med] | 4 | <.0001 | 0.0093 | 0.0033 | Synergy | 75±18 | 103±6 | 27±17 |
| Bacillus sp. [high] | Chitinophaga arvensicola [high] | 4 | <.0001 | <.0001 | 0.22 | Additive | 75±18 | 51±4 | 12±4 |
| Janthinobacterium sp. [low] | Pseudomonas sp. [low] | 4 | 0.3071 | 0.2451 | 0.0767 | No effect | 104±8 | 103±7 | 90±5 |
| Janthinobacterium sp. [low] | Pseudomonas sp. [med] | 4 | 0.6642 | 0.0851 | 0.2111 | No effect | 104±8 | 98±7 | 90±5 |
| Janthinobacterium sp. [low] | Pseudomonas sp. [high] | 4 | 0.3825 | 0.0001 | 0.1501 | Additive | 104±8 | 76±17 | 62±7 |
| Janthinobacterium sp. [med] | Pseudomonas sp. [low] | 4 | 0.0011 | 0.1367 | 0.036 | Synergy | 92±6 | 103±7 | 77±3 |
| Janthinobacterium sp. [med] | Pseudomonas sp. [med] | 4 | 0.0002 | 0.002 | 0.0059 | Synergy | 92±6 | 98±7 | 63±5 |
| Janthinobacterium sp. [med] | Pseudomonas sp. [high] | 4 | 0.34 | 0.0026 | 0.7584 | Additive | 92±6 | 76±17 | 72±9 |
| Janthinobacterium sp. [high] | Pseudomonas sp. [low] | 4 | <.0001 | 0.4186 | 0.282 | Additive | 24±40 | 103±7 | 3±1 |
| Janthinobacterium sp. [high] | Pseudomonas sp. [med] | 4 | <.0001 | 0.2957 | 0.3999 | Additive | 24±40 | 98±7 | 3±2 |
| Janthinobacterium sp. [high] | Pseudomonas sp. [high] | 4 | <.0001 | 0.0695 | 0.8982 | Additive | 24±40 | 76±17 | 3±1 |
| Janthinobacterium sp. [low] | Chitinophaga arvensicola [low] | 5 | <.0001 | <.0001 | 0.006 | Synergy | 70±5 | 67±7 | 56±4 |
| Janthinobacterium sp. [low] | Chitinophaga arvensicola [med] | 5 | <.0001 | <.0001 | 0.0088 | Synergy | 70±5 | 72±1 | 58±5 |
| Janthinobacterium sp. [low] | Chitinophaga arvensicola [high] | 5 | <.0001 | <.0001 | 0.0002 | Synergy | 70±5 | 71±4 | 67±5 |
| Janthinobacterium sp. [med] | Chitinophaga arvensicola [low] | 5 | <.0001 | <.0001 | 0.0373 | Synergy | 74±4 | 67±7 | 54±5 |
| Janthinobacterium sp. [med] | Chitinophaga arvensicola [med] | 5 | <.0001 | <.0001 | 0.6367 | Additive | 74±4 | 72±1 | 44±6 |
| Janthinobacterium sp. [med] | Chitinophaga arvensicola [high] | 5 | 0.0031 | 0.0008 | 0.0564 | Additive | 74±4 | 71±4 | 64±18 |
| Janthinobacterium sp. [high] | Chitinophaga arvensicola [low] | 5 | <.0001 | <.0001 | <.0001 | Synergy | 16±1 | 67±7 | 20±2 |
| Janthinobacterium sp. [high] | Chitinophaga arvensicola [med] | 5 | <.0001 | <.0001 | 0.0002 | Synergy | 16±1 | 72±1 | 11±5 |
| Janthinobacterium sp. [high] | Chitinophaga arvensicola [high] | 5 | <.0001 | <.0001 | 0.1412 | Additive | 16±1 | 71±4 | 4±1 |

Supplementary Table 2. Comparison of means of co-cultures. An ANOVA was used to determine if co-cultures additively inhibited *Bd*, synergistically inhibited *Bd* or facilitated *Bd*. Percent of *Bd* growth when grown each isolate alone or in co-culture with standard deviation are included.

| Combination | | P value | | |  | % *Bd* Growth | | |
| --- | --- | --- | --- | --- | --- | --- | --- | --- |
| Treatment 1 (T1) | Treatment 2 (T2) | T1 main effect | T2 main effect | Interaction | Biological effect | T1 | T2 | Co-culture |
| *Bacillus* | *Chitinophaga* | 0.13 | 0.0019 | 0.005 | Synergy | 131±55 | 92±52 | 2±2 |
| *Bacillus* | *Janthinobacterium* | 0.22 | 0.0002 | 0.004 | Synergy | 131±55 | 79±14 | 11±4 |
| *Bacillus* | *Pseudomonas* | 0.1317 | 0.0452 | 0.0028 | Synergy | 131±55 | 121±21 | 40±22 |
| *Pseudomonas* | *Janthinobacterium* | 0.3093 | 0.8971 | 0.0112 | Synergy; Facilitation | 71±22 | 77±7 | 113±16 |
| *Pseudomonas* | *Chitinophaga* | 0.0003 | 0.0033 | 0.1121 | Additive | 71±22 | 82±5 | 19±10 |
| *Janthinobacterium* | *Chitinophaga* | <.0001 | 0.0003 | 0.0123 | Synergy | 77±7 | 82±5 | 6±2 |
